# Regulating clinical trials in a resource-limited setting during the Ebola public health emergency in Sierra Leone

**DOI:** 10.1186/s13063-022-06416-4

**Published:** 2022-06-06

**Authors:** Onome T. Abiri, Abdulai J. Bah, Michael Lahai, Durodami R. Lisk, James P. Komeh, Joy Johnson, Wiltshire C. N. Johnson, Sheku S. Mansaray, Joseph Sam Kanu, James B. W. Russell, Fawzi Thomas, Murtada M. Sesay, Thomas A. Conteh, Alphan Tejan-Kella, Mohamed Sesay, Manal Ghazzawi, Brian Thompson, Sorie Conteh, Gibrilla Fadlu Deen

**Affiliations:** 1 Department of Pharmacology and Therapeutics, Faculty of Basic Medical Sciences, College of Medicine and Allied Health Sciences, University of Sierra Leone, Freetown, Sierra Leone; 2 Pharmacy Board of Sierra Leone, Freetown, Sierra Leone; 3 Department of Pharmaceutical Chemistry, Faculty of Pharmaceutical Sciences, College of Medicine and Allied Health Sciences, University of Sierra Leone, Freetown, Sierra Leone; 4 Department of Internal Medicine, Faculty of Clinical Sciences, College of Medicine and Allied Health Sciences, University of Sierra Leone, Freetown, Sierra Leone; 5 Department of Clinical Pharmacy, Faculty of Pharmaceutical Sciences, College of Medicine and Allied Health Sciences, University of Sierra Leone, Freetown, Sierra Leone; 6 Department of Community Medicine, Faculty of Clinical Sciences, College of Medicine and Allied Health Sciences, University of Sierra Leone, Freetown, Sierra Leone; 7 National Medical Supplies Agency, Freetown, Sierra Leone; 8 CitiGlobe Pharmacies Ltd, Freetown, Sierra Leone

**Keywords:** Clinical trial, Ebola, Vaccines, Therapeutics, Diagnostics, Expert committee, Medicine regulatory authority, Sierra Leone

## Abstract

Clinical trials during public health emergencies of novel medical products such as therapeutics and vaccines in resource-limited settings are daunting due to the limited capacity for regulatory assessment. Regulating clinical trials during the Ebola outbreak in Sierra Leone required expedited evaluation to identify medical products that could be promptly introduced to combat the epidemic in the absence of approved treatment or prevention. This article explored the decisions taken by the Pharmacy Board of Sierra Leone through its Expert Committee on Medicine Safety and Clinical Trials regarding clinical trials oversight during the Ebola epidemic and the lessons learned. This independent expert committee assessed and provided scientific opinions to the Pharmacy Board of Sierra Leone to inform approval of all clinical trials within 10–15 working days. We also requested for assisted review from the African Vaccine Regulatory Forum and support from the US Food and Drug Administration through a unilateral recognition and reliance memorandum of understanding. In addition, the Agency-ensured structures and systems were in place for reporting and reviewing adverse events and serious adverse events, management of biological samples, submission and review of progress reports, and good clinical practice inspections. Unfortunately, the Ebola epidemic revealed many weaknesses in the country’s clinical trials regulatory structure and processes. Government and partners should further offer more resources to build the clinical trial structures and systems so that the Agency will be better poised to handle future public health emergencies.

## Introduction

Sierra Leone was rebuilding its fragile health system after the gruesome armed conflict of 1991 to 2002 when the Ebola virus disease (EVD) epidemic struck [1]. This disease was unparalleled and unprecedented in terms of the rate and extent of spread across all the 16 districts in Sierra Leone, affecting routine healthcare services in health facilities as the epidemic progressed. Case fatality rate ranged from 30 to 90%, with an overall mortality of over 4000 [2–6].

During the outbreak, no approved therapeutics, biotherapeutics, or vaccines were available to manage and prevent EVD, as most were in pre-clinical or clinical trial stages. Clinical trials of novel medical products during public health emergencies in resource-constrained settings are daunting due to the limited capacity for regulatory reviews [7]. Applications for clinical trials during the EVD epidemic in Sierra Leone required accelerated assessment and approval to enhance access to life-saving products needed to fight the disease. This article provides the perspectives of the national medicine regulatory authority (NMRA), the Pharmacy Board of Sierra Leone (PBSL), and its Independent Expert Committee on Drug Safety and Clinical Trials regarding regulating clinical trials during the Ebola outbreak and the lessons learned for future considerations.

## Clinical trial oversight before the EVD outbreak

The PBSL lacked some of the basic clinical trial systems and processes as stipulated by the World Health Organization (WHO) Global Benchmarking Tool (GBT) for the evaluation of the national regulatory system of medical products before the outbreak [8]. Some of these include lack of comprehensive clinical trials guidelines defining the framework of clinical trials’ oversight, format and content of a clinical trial application (CTA), and the capacity to conduct GCP inspection. In addition, requirements for reporting adverse events or reactions, importation and destruction of investigational products (IPs), and recognition and reliance on scientific decisions from other mature NMRAs or international bodies were not in existence.

There was no independent expert committee on clinical trials with clearly defined composition and terms of reference (ToR) to support the review of CTAs and provide scientific opinions on clinical trial oversight activities. Furthermore, the PBSL was inadequately resourced with trained, experienced, and competent CTA assessors and GCP inspectors vested to execute clinical trial oversight activities fully. Standard operating procedures (SOPs) for clinical trial activities such as CTA assessment (clinical, quality, nonclinical, and biostatistics), GCP inspection, safety reporting and evaluation, progress reporting, and protocol amendment were not available.

## Clinical trial oversight during the EVD outbreak

### Reconstitution of PBSL expert committee on drug safety

Immediately after we recorded our first EVD case, PBSL reconstituted its Expert Committee on Pharmacovigilance by expanding its Terms of Reference (ToR) and membership to incorporate clinical trial activities. The membership’s expertise includes but is not limited to internal medicine, neurology, cardiology, pathology, epidemiology, biostatistics, quality assurance, quality control, pharmacology, and toxicology, categorised into clinical, quality, nonclinical, and biostatistics assessors.

The ToR included but was not limited to scientific opinions on clinical trials and medicines safety issues. In addition, the committee had the right to recommend to PBSL actions related to halting or suspending a clinical trial, Good Clinical Practice (GCP) inspection, and resolving issues or concerns related to pharmacovigilance and post-approval product safety, quality, and efficacy.

### Expression of interest and clinical trial applications of medical products received and assessed

To date, the PBSL has assessed ten repurposing applications for medical product registration and clinical trials, 21 clinical trials and compassionate use applications, and 18 amendments for the use of products to treat, prevent, and diagnose EVD.

Rudrapal et al. [9] stated that medicine repurposing or re-tasking identifies new therapeutic indications for discontinued, investigational, known, and approved drugs since traditional research and drug development is cost-intensive and prone to failure. Two drug repurposing applications were assessed, namely for registration and clinical trials. These applications were submitted with little or no scientific evidence to support their therapeutic claims in preventing or treating EVD. The products included nanosilver, ozone therapy, selenium, glutathione-200, doxycycline and garlic, 5-aminolevulinic acid, amiodarone, azithromycin, and sunitinib/erlotinib. Others were atorvastatin, clomiphene, enalapril, and irbesartan (Table 1).

**Table 1:** Repurposing applications for registration and clinical trials

Product	Purpose of application	Data available	Regulatory decision
Nanosilver	For the treatment of EVD	No	Objection
Ozone therapy	For the prevention and treatment of EVD	No	Objection
Selenium	For the prevention and treatment of EVD	No	Objection
Glutathione-200	For the prevention and treatment of EVD	No	Objection
Doxycycline and garlic	For the prevention and treatment of EVD	No	Objection
5-Aminolevulinic acid	For the prevention and treatment of EVD	No	Objection
Clomiphene, enalapril, and irbesartan	For the treatment of EVD	No	Objection
Clomiphene, enalapril, and irbesartan	For the treatment of post EVD symptoms	No	Objection
Amiodarone	Clinical trial application submitted for the treatment of EVD	No	Objection
Azithromycin, sunitinib/erlotinib, atorvastatin, and irbesartan	Clinical trial application submitted for the treatment of EVD	No	Objection

*EVD* Ebola virus disease

Five therapeutic clinical trial applications for EVD were also received and evaluated. These included convalescent whole blood (CWB), convalescent plasma (CP), ZMapp®, TKM-130803, and Brincidofovir, which the sponsor later withdrew due to logistical reasons (Table 2).

**Table 2:** Clinical trial and compassionate use applications for therapeutics, vaccines, and diagnostics

Intervention	Product	Phase/study type	Short title	Regulatory decision
Therapeutics	Convalescent whole blood (CWB)	2/3	Effectiveness of CWB in the treatment of consented EVD patients	No objection
	Convalescent plasma (CP)	2/3	Effectiveness of CP in the treatment of consented EVD patients	No objection
	ZMapp®	2/3	Safety and efficacy of ZMapp	No objection
	TKM-130803	2/3	Safety and efficacy of TKM 130803	No objection
	Brincidofovir	2	Safety and efficacy of brincidofovir	CTA withdrew by the sponsor
	FX-06	Compassionate use	Compassionate use of FX-06	No objection
	ZMapp®	Compassionate use	Expanded access programme for ZMapp	No objection
Vaccines	rVSV-ZEBOV-GP	2/3	Safety and immunogenicity of rVSV-ZEBOV-GP	No objection
	Ad26.ZEBOV/MVA-BN-Filo	2/3	Safety and immunogenicity of Ad26.ZEBOV/MVA-BN-Filo	No objection
	Ad5-EBOV	2	Safety and immunogenicity of Ad5-EBOV	No objection
	Ad26.ZEBOV/MVA-BN-Filo/rVSV-ZEBOV-GP	2	Safety and immunogenicity of the three vaccines	No objection
	Ad26.ZEBOV/MVA-BN-Filo	2	Long-term safety and immunogenicity of Ad26.ZEBOV/MVA-BN-Filo	No objection
	Ad26.ZEBOV/MVA-BN-Filo	2	Safety and immunogenicity in infants 4-11 months	No objection
	Ad26.ZEBOV	2	Safety and immunogenicity in children previously vaccinated with the two doses of Ad26.ZEBOV/ MVA-BN-Filo	No objection
	rVSV-ZEBOV-GP	Compassionate use	Ring vaccination compassionate use of rVSV-ZEBOV-GP	No objection
	Ad26.ZEBOV/MVA-BN-Filo	Compassionate use	Deployment and effectiveness of Ad26.ZEBOV/MVA-BN-Filo	No objection
Rapid diagnostic tests	Zalgen recombinant RDT	Validation	Evaluation of the performance of Zalgen	No objection
	Biocartis RDT	Validation	Evaluation of the performance of Biocartis	Objection
	Cepheid RDT	Validation	A validation study of the performance of Cepheid RDT	No objection
	Micro BB RDT	Validation	Evaluation of the performance Micro BB RDT	No objection
	BSL-2 Assay RDT	Validation	Evaluation of the performance of BSL-2 assay RDT	No objection

*CWB* convalescent whole blood, *CP* convalescent plasma, *rVSVΔG-ZEBOV-GP* recombinant vesicular stomatitis virus vector carrying Zaire Ebola virus glycoprotein, *Ad26.ZEBOV* adenovirus type 26 vector-based vaccine expressing Zaire Ebola virus glycoprotein, *MVA-BN®-Filo* recombinant, modified vaccinia Ankara (MVA) vector-based vaccine, encoding glycoproteins from Zaire Ebola virus, Sudan virus, and Marburg virus, and nucleoprotein from the Tai Forest virus, *Ad5-EBOV* adenovirus type 5 vector-based Ebola virus disease vaccine

Vaccine trials of IPs, namely recombinant vesicular stomatitis virus vector (rVSVΔG-ZEBOV), human adenovirus type 26 (Ad26.ZEBOV), Modified Vaccinia Ankara Bavarian Nordic (MVA-BN®-Filo), and human type 5 adenovirus (Ad5-ZEBOV) EVD glycoprotein vaccines were approved and investigated. Some Ebola rapid diagnostics tests (RDTs) such as Zalgen, Cepheid, Micro BB, and BSL-2 assay were evaluated and validated. Another RDT called Biocartis was later disapproved because the investigator failed to meet regulatory requirements (Table 2).

Compassionate use is a pre-authorisation process in which patients with life-threatening conditions are granted access to investigational medical products (IMPs) such as vaccines and therapeutics outside a clinical trial [10, 11]. Medical products approved and deployed for compassionate use included FX-06, rVSVΔG-ZEBOV-GP vaccine, and ZMapp (Table 2). However, Mapp Biopharmaceuticals withdrew the Zmapp application in November 2020 due to the approval by the US Food and Drug Administration (FDA) of Inmazeb (atoltivimab, maftivimab, and odesivimab) and Ebanga (Ansuvimab) in 2020.

### Timelines for review of CTA

The timeline stipulated by the PBSL guideline for conducting clinical trials from receipt to approval of an application is 60 working days for therapeutics, vaccines, and medical devices. However, PBSL conducted expedited reviews within 10–15 working days. This was done to facilitate product development and expedite clinical trials to determine therapeutics, vaccines, and medical devices that might be useful in managing, preventing, and diagnosing EVD. In addition, the Agency held pre-submission meetings with sponsors to understand the regulatory process and fast-track submissions, reviews, and approvals.

### Regulatory considerations for the review and approval of CTA

The PBSL evaluated all clinical trial applications using PBSL clinical trial and GCP Guidelines. Other guidelines utilised include the International Conference on Harmonisation (ICH) technical procedures. These include ICH Quality (Q) guidelines (Q1, Q2, and Q5), ICH Safety (S) guidelines (S1-S8), ICH efficacy (E) guidelines (E2-E3, E6, E8-E9), ICH multidisciplinary (M) guideline (M3), and WHO nonclinical and clinical evaluation of vaccines guidelines [12–14]. These guidelines provide the highest scientific norms for designing, conducting, and reporting clinical research and ensuring study participants’ safety and well-being.

The following elements were considered crucial for a study vaccine or therapeutic for clinical trials. These included a demonstration of efficacy in non-human primates (NHP) and a rationale for the proposed dosing in humans regarding exposures shown to be effective in suitable models. In addition, the PBSL considered the safety assessment of the product at the exposure level proposed for treatment or prevention of the disease and information on the chemistry, manufacturing, and control of the IMP. All trials were required to recruit a local principal investigator as stipulated in the national clinical trials guideline and regulations. Submission of CTA to PBSL usually is accompanied by ethics approval from the Sierra Leone Ethics and Scientific Review Committee (SLESRC). However, parallel submissions during the Ebola were accepted. In addition, sponsors were required to register their studies with the Pan African Clinical Trial registry (PACTR) to inform patient care whether authors decide to report or journals decide to publish the trial outcomes [15]. Table 3 shows queries and observations from reviews sent to sponsors for feedback.

**Table 3:** Observations sent to sponsors from PBSL’s review of CTA and amendments

Areas of review	Observations
**General requirements**	▪ Trial not registered with a PBSL approved clinical trial registry
	• No or incomplete DSMB charter, including membership, the charter of work, study review criteria/stopping rules, curriculum vitae, and conflict of interest details
	• Completed PBSL CTA application form not available
	• Pharmacy manual not provided
	• No contractual agreement between the sponsor and principal investigator
	• No local principal investigator was recruited
**Clinical protocol**	• No specification for assessment of efficacy and safety
	• No criteria for participant selection
	• Unclear study endpoints
	• Studies containing no local sub-investigators and study pharmacists
	• Favourable opinion from the SLESRC not available
	• A description of the design of the trial to be conducted was not provided
	• A description and justification of the trial treatment and the dosage and dosage regimen of the investigational product were not provided
	• A detailed description of the “stopping rules” or “discontinuation criteria” is unavailable.
	• Valid insurance certificate for the study duration that must be provided before study initiation is not available.
	• The informed consent information sheet does not have details of the Chairman of the Sierra Leone Ethics and Scientific Review Committee for participants to contact if they have ethical issues.
	• Procedures for monitoring subject compliance not provided
	• The sponsor intends to conduct a phase 2 clinical trial, but the phase 1 trial report was not available
	• No details of causality assessment parameters and serious adverse events/reaction toxicity grading such as those for haematology and biochemistry
	• No evidence of GCP training for the principal investigator and other key staff
	• No details of IP data handling and recording keeping
**Quality review**	• Process validation protocol and report were not available
	• Evidence of Good Manufacturing Practice compliance for the manufacturing site(s) of IP and excipients not available
	• Analytical Procedures and batch analyses for IP and excipients not provided
	• No analysis report of reference standards, including test methods, acceptance criteria, and results
	• Post-approval stability protocol and stability commitment for ongoing stability studies of IP not provided
	• Sample of labels not available
	• The product dossier for the placebo was not provided
	• The parameters, test methods, specifications or acceptance criteria and results for the pre-master virus seed are not available
	• Genotypic and phenotypic characterisation of the master virus seed was not available
	• No read-outs or tracings for characterisation of impurities
	• No tracings or read-outs for analytical method validation
**Biostatistics review**	• Criteria for the termination of the trial are not available
	• Timing of any planned interim analysis though, was planned not provided
	• Incomplete statistical analysis plan submitted before data lock, with no authors’ name and signature, version number and date, and no inclusion and exclusion criteria
**Nonclinical review**	• Investigator’s brochure not provided
	• No developmental and reproductive toxicity data to support use in pregnancy

*PBSL* Pharmacy Board of Sierra Leone, *DSMB* Data Safety Monitoring Board, *CTA* clinical trial application, *SLESRC* Sierra Leone Ethics and Scientific Review Committee, *GCP* Good Clinical Practice, *IP* investigational product, *PI* principal investigator

### Progress reporting and GCP inspections

Progress reports were required from investigators from the date of commencement of the trial in the recommended format and timeline as stated in the PBSL clinical trials guideline. These include quarterly progress, annual, interim, trial site close-out, and final clinical study reports. In addition, sponsors submitted data and safety monitoring board (DSMB) reports on request.

The Pharmacy Board of Sierra Leone conducted Good Clinical Practice (GCP) inspections using the ICH GCP (R2) guidelines [16]. Trial sites were inspected at least once during the life span of the study, which entails a review of essential documents, facilities, records, and other resources like computer systems and equipment. In addition, the Pharmacy Board established standard operating procedures (SOP) and timelines for preparing, coordinating, conducting, and reporting inspection findings. Table 4 shows some common GCP findings observed during inspections, including but not limited to quality assurance issues with data recording, protocol deviations, problems with adverse event reporting, and failure to submit progress reports.

**Table 4:** Common GCP inspection findings

Areas inspected	GCP inspection findings
**Operational resources**	• Delegation log incomplete or not available
	• Training records for some staff are unavailable
	• Normal reference ranges were not updated
	• Computer validation protocol and report were not available
	• Trial initiation monitoring report not available
**Trial master file**	• Incomplete participant screening and enrolment log
	• Case report forms not filled completely
	• Curriculum vitae for key staff not available
	• Incomplete informed consent forms
	• Contract agreements were not available
**Conduct of the trial**	• Issues with participant eligibility logs
	• Problems with participant identification logs
	• Emergency trolley not secured and under lock and key
**Management of trial of sponsor/CRO**	• Issues with protocol deviation management
	• Quarterly progress report not available
	• Monitoring plan not available
	• No corrective action plan and corrective action report as a result of a monitor’s visit
**Safety reporting**	• SAEs not reported
	• Development Safety Update Reports not submitted
	• SAEs are not processed according to the SOPs and the PBSL guideline
**Investigational product/pharmacy**	• IWRS validation report not available
	• Inadequate IP accountability
	• Logbooks not available
	• Pharmacy is not adequately designed and equipped.
**Clinical data management**	• Issues with data entry and verification
**Source data verification**	• Problems with source data verification
**Laboratory**	• Laboratory normal ranges/references not updated
	• Laboratory analytic plan not signed and endorsed
	• Equipment qualification reports were not available
	• Logbooks not available
**Quality management system**	• Obsolete SOPs in use
	• Quality assurance/audit report not available
	• Some SOPs were not available
	• SOPs and documents in a foreign language

*SAE* serious adverse event, *PBSL* Pharmacy Board of Sierra Leone, *IWRS* Interactive Web Response System, *IMP* investigational medical product, *SOPs* standard operating procedure, *GCP* Good Clinical Practice

### Safety monitoring in clinical trials

Adequate systems and processes for monitoring patient safety are often a challenge in resource-constrained settings [17]. During a clinical trial, adverse events were identified, collected, and analysed to meet regulatory requirements for the protection of patients and enable sufficient safety characterisation of the IP [18, 19].

The national requirements for safety reporting include submitting serious adverse events (SAE), follow-up reports, development safety update reports (DSUR), and reports from foreign sites. The SAEs received immediate medical attention and were reported within 48 h. The SAE reporting forms were an adapted version of the Council for International Organisation of Medical Sciences (CIOMS) 1 to enable causality assessment. In addition, all fatal cases were supposed to be accompanied by an autopsy report. However, verbal autopsy reports were submitted because of the virulent nature of the virus in the dead. The WHO verbal autopsy instrument was utilised for this purpose, and PBSL required that the interviews be done by a medical doctor and verified by another medical doctor. The Agency has received over 100 SAEs and three serious unexpected suspected adverse reactions (SUSARs) from the different clinical trials. In addition, PBSL issued four Dear investigator letters to reclassify causality assessment from unrelated to related and change of toxicity severity grading. Other information such as laboratory tests (haematology and biochemistry), verbal autopsy reports, and follow-up reports were required to enable thorough safety assessments.

### Regulatory recognition, reliance, and joint CTA reviews

During the EVD outbreak, the PBSL faced an additional burden amidst its meagre resources to review, approve, and monitor CTAs of novel therapeutics, vaccines, and biotherapeutics.

As a result, PBSL requested an African Vaccine Regulatory Forum (AVAREF)-assisted review for the Janssen Ad26.ZEBOV and MVA-BN-Filo vaccines. This platform brought together experts of NMRAs, and national ethics committees from the USA, Europe, Canada, Switzerland, Ghana, and Nigeria to ensure expedited review of the application in a shorter timeframe than the usual 60 days [20]. Joint review meetings were also held between PBSL and the SLERSC to discuss the new technological platforms of the vaccines and data availability for justifying some repurposed products such as Amiodarone. In addition, PBSL utilised a unilateral collaboration and recognition through a signed memorandum of understanding (MoU) with the USFDA for CTAs received for the rVSV-ZEBOV-GP vaccine and Zmapp. The ultimate goal was to avoid repetition, focus limited resources on critical areas, and expedite access to novel medical products.

### Biological samples management and material transfer agreement

Biobanking is crucial in ensuring biosafety and biosecurity and can promote valuable health research that may lead to significant societal benefits. However, collecting, storing, and transferring human samples has challenges, mainly when samples are transported from low-income countries like Sierra Leone to laboratories in high-income countries [21].

In the wake of the Ebola epidemic, several local and international institutions were involved in the unauthorised export of Ebola samples [22]. All applications that we received for clinical trials involve biological sample transfer to laboratories abroad for future research purposes and retention. As a result, PBSL, in collaboration with the Ministry of Health and Sanitation (MoHS) and the Office of National Security, put in place a system for approval and issuance of an export permit. The PBSL’s requirement for material transfer authorisation stipulates that all applications must be accompanied by evidence of informed consent and a material transfer agreement (MTA). Applicants were also expected to provide an annual update on the results obtained from biological samples exported from Sierra Leone. The feedback provided was concerning the results of immunological analyses performed outside of Sierra Leone.

## Current status of clinical trials oversight in Sierra Leone after the Ebola epidemic

In 2018, PBSL requested technical support in clinical trials and pharmacovigilance oversight from the Paul Ehrlich Institute (PEI) in Germany. The PEI did a gap analysis using the WHO GBT, identified areas for improvement, and created institutional development plans (IDPs) with the Agency. The PEI provided technical and financial resources to address the IDPs. These include the provision of resources for the capacity development of our staff in CTA review (pre-clinical, clinical, and quality) and GCP inspection. In addition, we received support for developing a clinical trial regulation, updating, and creating operational guidelines, SOPs, and templates based on the WHO GBT. As a result, we have adapted and used the AVAREF clinical trials resources such as the CTA checklist and form, GCP inspection guideline and biostatistics, clinical, pre-clinical, and quality review templates. The Food and Drug Authority of Ghana, through their Regional Centre of Regulatory Excellence (RCORE) clinical trial fellowship and the Health Canada, also provided some capacity-building support.

## Lessons learnt for future public health emergencies like the COVID-19 pandemic

The Ebola epidemic provided opportunities to identify gaps in our regulatory processes and mapped new ways to improve them. Therefore, we have adopted the AVAREF model of expedited review and started implementing a new policy on recognition and reliance. Collaboration with well-resourced NMRAs and AVAREF-assisted reviews helped reduce workload and expedited product development and the introduction of life-saving medical products. These collaborations also allowed us to share knowledge, experience, and best practices. The unilateral or multilateral information sharing provided an open channel of communication which was crucial in responding rapidly to public health emergencies of international concern. There is a need to further build technical capacity in GCP inspection and CTA evaluation, focusing on specialisations such as nonclinical, clinical, biostatistics, and quality reviews. Penal sanctions should be enforced for individuals or local and international health instructions that violate national regulatory requirements. The involvement of local investigators and other study team members in the trials was pivotal to the operational success of these studies. Pre-submission meetings provided the environment for sponsors and PBSL to discuss the contents of the application and answer questions. This reduced delays in meeting timelines and removed unnecessary bottlenecks in the approval process.

## Conclusion

Some of the vaccines tested in Sierra Leone, such as Ervebo, Zabdeno, and Mvabea Ebola prevention vaccines, have been granted regulatory approval by NMRAs such EMA, USFDA, and PBSL. These vaccines have also been deployed in the Democratic Republic of Congo to help curb the recent EVD outbreaks.

Regulation of clinical research in resource-limited settings like ours during the Ebola outbreak spotlighted gaps and challenges in the clinical trial regulatory systems and processes. Collaboration with well-resourced NMRAs and regional scientific bodies such as AVAREF promoted expedited review and timely introduction of life-saving medical products. In addition, we leveraged on matured NMRAs that provided technical support for capacity building to improve clinical trial regulatory functions of the PBSL. Nevertheless, there is a need for our government, local, and international partners to further invest in clinical trial regulatory systems strengthening to enable the PBSL to handle clinical trial applications both in and without public health emergencies.

## Data Availability

Not applicable.
